# Comparison of the surgical efficacy of total hip replacement versus hemiarthroplasty in the treatment of femoral neck fractures in elderly patients with sarcopenia

**DOI:** 10.1371/journal.pone.0321070

**Published:** 2025-04-01

**Authors:** Zhaoyang Yin, Zhuzhi Zhu, Chao Wang, Xiaolong Jia, Xiuqiang Zou

**Affiliations:** 1 Department of Orthopedics, the Affiliated Lianyungang Hospital of Xuzhou Medical University (The First People’s Hospital of Lianyungang), Lianyungang, China; 2 Department of Orthopedics, Nanjing Lishui People’s Hospital, Zhongda Hospital Lishui Branch, Southeast University, Nanjing, China; 3 Department of Orthopedics, the Affiliated Jiangning Hospital with Nanjing Medical University, Nanjing, China; Harvard Medical School, UNITED STATES OF AMERICA

## Abstract

**Background:**

Total hip arthroplasty (THA) and hemiarthroplasty (HA) are common surgical procedures for femoral neck fracture (FNF) in elderly patients; however, optimal treatment options remain controversial. Currently, limited research has compared the effectiveness of THA versus HA, specifically in patients with FNF and sarcopenia.

**Methods:**

This retrospective study included data from 109 patients who fulfilled the inclusion criteria for the period between January 2015 and December 2017. Among these, 48 underwent THA, and 61 underwent hip arthroplasty (HA). The cross-sectional area (cm^2^) of muscle tissue at the pedicle level of the 12th thoracic vertebra (T12) was measured using chest computed tomography. The skeletal muscle index (SMI) was calculated by dividing the cross-sectional area of the muscle at the T12 pedicle by height squared. Sarcopenia was diagnosed when grip strength and SMI values were below the diagnostic cut-off value. Various factors were compared, including age, sex, SMI, body mass index (BMI), perioperative surgery-related indicators, postoperative 5-year survival, satisfaction, complication, and re-revision surgery rates between the 2 groups.

**Results:**

There were no statistically significant differences between the THA and HA groups in terms of age (*P* =  0.227), sex (*P* =  0.870), SMI (*P* =  0.946), BMI (*P* =  0.310), postoperative time to ambulation (*P* =  0.803), length of hospitalization (*P* =  0.777), postoperative visual analog scale score (*P* =  0.933), and postoperative Harris score (*P* =  0.379). At the 5-year follow-up, there were no statistical differences in patient survival rate (*P* =  0.896), satisfaction (*P* =  0.945), incidence of complications (*P* =  0.796), or re-revision rate (*P* =  0.807). Patients who underwent THA had significantly longer operative times (P =  0.000) and larger surgical incisions (P =  0.000). They also experienced greater blood loss (P =  0.000) and blood transfusion volumes (P =  0.017), as well as increased hemoglobin (P =  0.000) and albumin (P =  0.000) loss. Furthermore, patients who underwent THA incurred higher surgical costs (P =  0.000).

**Conclusion:**

THA and HA demonstrated comparable effectiveness and outcomes in patients with FNF and sarcopenia. HA was a less invasive and more cost-effective surgical option, making it the preferred choice.

## 1. Introduction

Femoral neck fracture (FNF), a type of hip fracture, is often referred to by orthopedic surgeons as “the last fracture in the strange journey of life”, which significantly and negatively impacts longevity and quality of life of the elderly. Studies have reported that the 30-day mortality rate of FNF is 3–10%, and the 1-year mortality rate can reach 30% [1–3]. One-quarter of patients require long-term family or nursing home care before they can live independently, and one-half struggle to fully return to pre-fracture activities [4]. Despite tremendous efforts to understand and treat this condition, the incidence of FNF remains high due to accelerated trends in global aging in recent decades. The risk for FNF doubles every decade after 50 years of age. One statistic indicates that the incidence of FNFs is projected to reach 6.3 million by 2050 [5], thus placing a heavy burden on healthcare systems. Falls and motor vehicle accidents are the most important causative factors of FNFs. Primary or secondary osteoporosis is a risk factor for FNFs [6,7]. Recent studies have shown that reduced muscle strength is a risk factor for FNF[8,9].

The term “sarcopenia” originates from the Greek words *sarx* (meat) and *penia* (poverty), and was first coined by Irwin Rosenberg in 1988 [10]. Sarcopenia is a disease characterized by progressive and systemic loss of skeletal muscle mass and strength. Sarcopenia is a debilitating condition that often results in falls, fractures, decreased quality of life, hospitalization, and even death [11–14]. As population aging continues to accelerate worldwide, the incidence of sarcopenia continues to increase. Statistics have shown that 13% of the global population is affected by sarcopenia [15], with an incidence ranging from 10% to 16% among those >  60 years of age [16], 1% to 29% among community-dwelling older adults, and 14%–33% among long-term care residents [17,18]. As age increases, healthy adults lose approximately 8% of their muscle mass every decade, starting from 40 years of age and, after 70 years of age, the muscle loss rate accelerates to 15% every decade [19,20]. The European Working Group on Sarcopenia in Older People (EWGSOP) uses muscle strength, muscle quantity/quality, and physical performance as indicators of the severity of sarcopenia [21]. In clinical settings, muscle mass and function measurement methods include computed tomography (CT), magnetic resonance imaging, dual-energy X-ray absorptiometry, and bioelectrical impedance analysis. Sarcopenia is one of the most common comorbidities among those who experience FNFs [22]. Trauma and bed confinement contribute to decreased muscle mass and function in patients with FNF. Approximately 5%–6% of muscle mass is lost in the first year after a hip fracture, and 28% of patients are unable to ambulate normally 12 months after surgery [22].

Arthroplasty, whether total hip arthroplasty (THA) or hemiarthroplasty (HA), has become the treatment of choice for elderly patients with FNF due to lower complication rates and significant improvements in hip function. The advantages of THA include higher resistance to joint wear and a lower revision rate; however, the disadvantages are also obvious, including greater surgical trauma and higher hospitalization costs. HA has the advantages of less surgical trauma, shorter operative duration, lower technical requirements, and lighter economic burden. The main disadvantages of HA include poor wear resistance and high revision rates [23–26]. As such, the optimal treatment for elderly patients with FNFs remains controversial. Medical comorbidities, cognitive status, limb function, and the physical demands of daily living are factors that orthopedic surgeons consider when selecting the most appropriate surgical approach. Studies have shown that THA is superior to HA and may be the preferred surgical procedure for those >  75 years of age; however, some investigators have raised objections [27]. Sarcopenia is a debilitating disease that is often observed in elderly patients who experience FNFs. However, whether this affects the choice of THA or HA has not yet been reported. The purpose of the present study, therefore, was to compare the outcomes of THA versus HA in elderly patients with FNF and sarcopenia, and to determine a better strategy by analyzing the differences in key indicators, including surgery-related indicators, blood transfusion, decreases in hemoglobin and albumin levels, postoperative recovery indicators, treatment costs, survival rate, satisfaction, and the incidence of complications, to provide a reference for selecting the most appropriate surgical treatment plan.

## 2. Methods

### 2.1 Ethics approval and patient consent

This study was approved in 2014 by the Ethics Committee of Jiangning Hospital Affiliated to Nanjing Medical University (Jiang Su Sheng, China; No. 201401015), and conducted in accordance with the Declaration of Helsinki and the International Conference on Harmonization Tripartite Guidelines on Good Clinical Practice. Informed written consent was obtained from all patients.

### 2.2 Study design

This single-center, retrospective, non-randomized, 5-year follow-up study reviewed and analyzed elderly patients with FNF who fulfilled the inclusion criteria and were admitted to the Department of Orthopedics, Jiangning Hospital Affiliated to Nanjing Medical University, between January 2015 and December 2017. Starting from January 2015, to date, we have collected clinical data from all participants with the knowledge and authorization of all participants. Strict inclusion and exclusion criteria were used to reduce data heterogeneity and patient selection bias. Demographic factors and primary outcome measures included age, sex, skeletal muscle index (SMI), body mass index (BMI), operative duration, intraoperative blood loss, visual analog scale (VAS) score, Harris score, incidence of complications, postoperative time to ambulation, length of hospitalization, hospitalization costs, satisfaction, and survival rate in the 5th year after surgery. Complications included incisional fat liquefaction and infection, deep vein thrombosis of the lower limbs, prosthetic dislocation, loosening or subsidence, periprosthetic fractures, and heterotopic ossification.

The inclusion criteria were as follows: non-cardiocerebrovascular accident; 70–85 years of age; FNF caused by a fall (non-violent fragility fracture); Garden classification type III or IV; no severe, uncontrolled hypertension, diabetes, coronary heart disease, or cerebral infarction; no bony and structural abnormalities around the hip joint; and unilateral FNF.

The exclusion criteria were as follows: age >  85 or <  70 years; history of hip surgery; mental symptoms precluding cooperation with treatment; failure to provide informed written consent; lower limb dysfunction, combined with serious medical disease(s), such as liver and kidney dysfunction, hyperthyroidism, severe hyperlipidemia; comorbid rheumatoid arthritis or osteoarthritis; pathological fracture; underwent previous non-artificial total hip replacement surgery; loss to follow-up; and did not undergo chest CT examination.

### 2.3 Diagnostic procedure for sarcopenia

**Fig 1:**
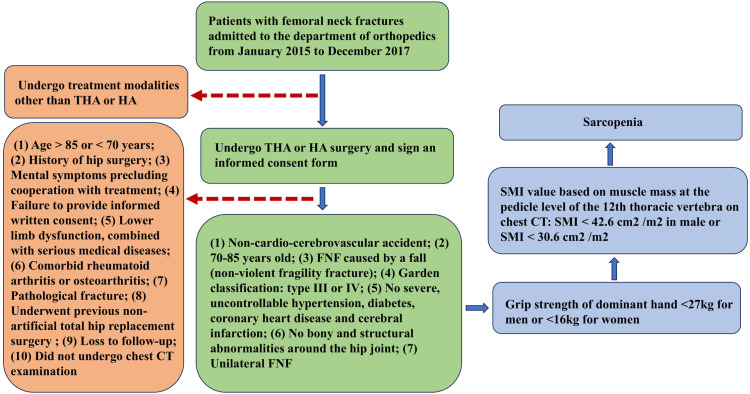
Flowchart of diagnosis of sarcopenia in patients included in the study.

Sarcopenia was diagnosed based on guidelines recommended by the EWGSOP in 2018 [28]. An electronic grip strength instrument (Kangdu, Guangdong, China) was used to measure the grip strength in the dominant hand (maximum value among 3 measurements). SMI was calculated when the grip strength value was lower than the diagnostic standard (males, <  27 kg; females, <  16 kg). Images were analyzed using PACS version 3.6 software (Philips, Hamburg, Germany). The total areas of the erector spinae, latissimus dorsi, internal oblique, external oblique, rectus abdominis, external intercostal muscles, and intercostal muscles were measured at the pedicle level of the 12th thoracic vertebra using chest CT. SMI was calculated by dividing the sum of the muscle areas by the patient’s height squared (cm^2^/m^2^). Patients were blinded to the 2 independent measurements. Sarcopenia was diagnosed as SMI <  42.6 cm^2^/m^2^ in males or <  30.6 cm^2^/m^2^ in females [29]. A diagnostic flow-diagram for sarcopenia among patients included in this study is presented in Fig 1.

### 2.4 HA and THA

#### Perioperative protocol.

All patients underwent bilateral hip radiography, chest CT, heart and lower limb vascular ultrasonography, routine blood tests, coagulation function tests, and biochemical index examinations before surgery. All surgeries were performed by the same team of physicians. Under general anesthesia, patients were positioned lateral decubitus and surgery was uniformly performed using the posterolateral Gibson approach. Based on intraoperative continuous data, an appropriate Zimmer Biomet (Warsaw, IN, USA) hip prosthesis (non-cemented bioprosthesis) was selected. The drainage tube was removed within 24 h after surgery, and rivaroxaban (Bayer, Berlin, Germany) was administered for anticoagulation for 35 days after surgery. Isometric quadricep contractions and ankle pump exercises were initiated 6 h after surgery. Knee flexion and straight leg raises commenced 1 day after surgery. On postoperative day 2, the patient was instructed to stand gradually and walk using a walking aid.

#### Rehabilitation plan.

The affected limb was elevated on the day after surgery to promote circulation. Full weight-bearing could be performed if the patient was able to tolerate the pain, with “go-to-ground” activities 3 to 4 times per day and walk with crutches for 5 min each time. The “go-to-ground” training involves the patient executing several exercises under the supervision of a rehabilitation physician. These exercises include shifting the center of gravity to the lower limb on the operated side, standing on one foot using the operated lower limb, alternately taking small steps in place, and alternately raising the leg in place. On postoperative day 3, passive functional exercise machine training could be adopted. After hospital discharge, follow-up telephone calls or outpatient follow-up visits were conducted.

#### Procedures.

**Fig 2.**
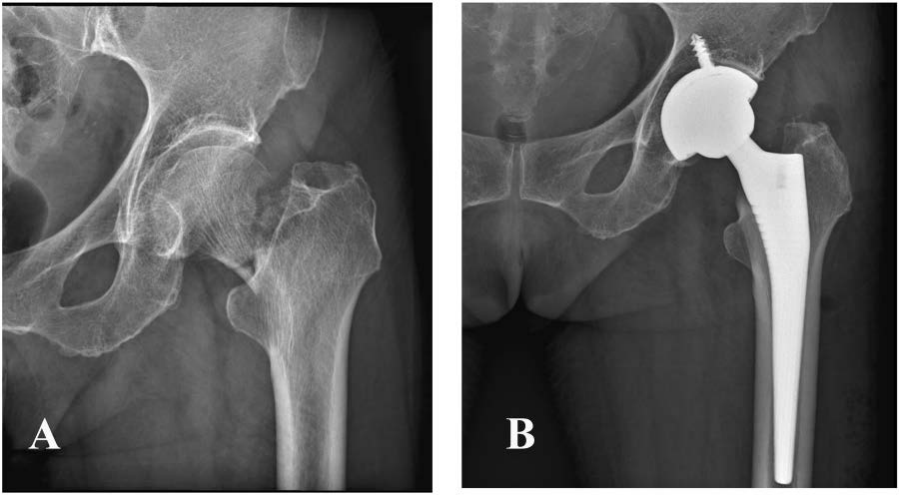
X.ray of a patient with a femoral neck fracture undergoing THA. Female, 80 years old, diagnosed with femoral neck fracture with sarcopenia (A) Preoperative X-ray (B) Postoperative X-ray.

*THA group*: A T-shape incision was performed to expose the patient’s hip joint. After removal of the femoral head, the neck was trimmed. The femoral medullary canal was enlarged, the acetabulum cleared, and a suitable hip prosthesis was installed. Representative radiographs before and after surgery are presented in Fig 2.

**Fig 3.**
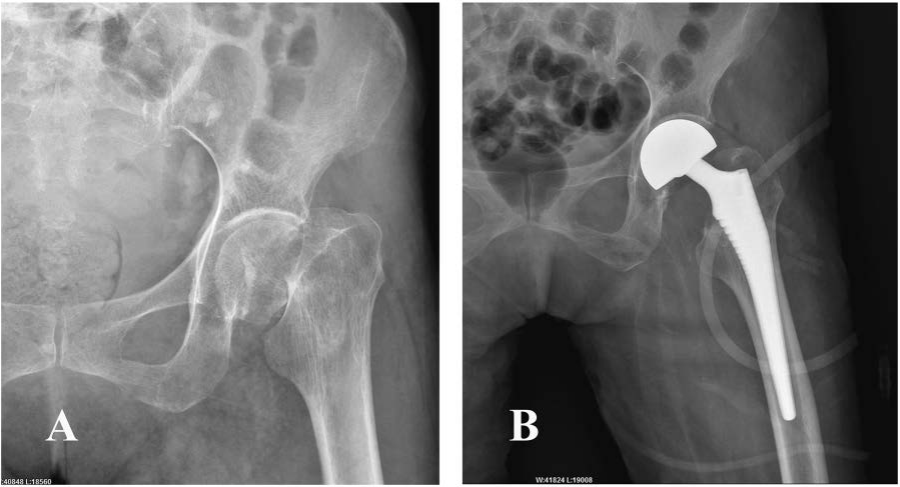
X.ray of a patient with a femoral neck fracture undergoing HA. Female, 76 years old, diagnosed with femoral neck fracture with sarcopenia (A) Preoperative X-ray (B) Postoperative X-ray.

*HA group*: After incising the joint capsule, the femoral head, neck, and base were exposed. The femoral head and femoral neck were removed, and the femoral medullary cavity was expanded. Subsequently, a suitable femoral head prosthesis is installed. Representative radiographs before and after surgery are presented in Fig 3.

### 2.5 Statistical analysis

Statistical analyses were performed using SPSS version 26.0 (IBM Corp., Armonk, NY, USA). This study had a retrospective cohort design. Based on the primary observed outcome indicators and the previous literature, an α =  0.05 (two-tailed) and 1–β =  0.80 were established. The minimum sample size for each group was calculated using PASS version 15 (NCSS LLC, Kaysville, Utah, USA), with adjustments made to increase the sample size to accommodate specific circumstances. A paired sampling method was used to select corresponding cases from the THA and HA groups, ensuring that they were as similar as possible in terms of age and sex. After estimating the minimum sample size, the study included participants and identified differences in age and gender. Subsequently, a paired sampling method based on these demographics was employed, and the sample size was increased to ensure that age and gender were as similar as possible between the experimental and control groups. Numerical variables are expressed as mean ±  standard deviation (SD). Continuous data were compared using the *t*-test, and numerical data were compared using Pearson’s χ^2^ test or continuity corrected χ^2^ test. Levene’s method was used to test for variance. When the variances were equal, an independent samples *t*-test was used to compare the 2 datasets. Conversely, when the variances were unequal, a corrected *t*-test was used to assess whether significant differences existed between 2 datasets. The Kaplan–Meier (KM) method was used for survival analysis, while the log-rank test was used to compare survival rates between the 2 patient groups. Differences with *P* <  0.05 were considered to be statistically significant.

## 3. Results

### 3.1 General characteristics of the THA and HA groups

According to the inclusion and exclusion criteria, 3 patients in the THA group were lost to follow-up; more specifically, 2 could not be contacted due to changes in contact information, and 1 refused to participate in the study. In the HA group, two patients were lost to follow-up due to changes in contact information, which made them unreachable. The general characteristics of the 2 patient groups are summarized in Table 1. In total, 48 and 61 patients underwent THA and HA, respectively. There were no significant differences in age (*P* =  0.227), sex (*P* =  0.870), SMI (*P* =  0.946), or BMI (*P* =  0.310) between the 2 groups.

**Table 1:** General characteristics of patients in two groups.

	THA (n = 48)	HA (n = 61)	*t* or *χ*^*2*^	*p* value	Cohen’s d/Phi	95%CI/OR(95%CI)
Age (*x ± s*, years)	78.04 ± 4.131	79.10 ± 4.784	−1.215	0.227	0.118	−1.057(−2.781,0.668)
Gender			0.027	0.870	−0.016	0.939(0.439,2.007)
Male	22	27				
Female	26	34				
SMI (*x ± s*, cm^2^/m^2^)	27.106 ± 6.676	27.193 ± 6.602	−0.068	0.946	0.0065	−0.08719(−2.62505,2.45066)
BMI (*x ± s*, kg/m^2^)	20.887 ± 5.017	19.847 ± 5.483	1.020	0.310	0.098	1.03996(−0.98111,3.06103)

THA: Total hip arthroplasty

HA: Hemiarthroplasty

SMI: Skeletal muscle index

BMI: Body mass index

OR: Odds Ratio

### 3.2 Comparison of perioperative surgery-related indicators

The perioperative surgery-related indicators of the 2 patient groups are summarized in Table 2. Statistical analysis revealed no significant differences in postoperative pain or recovery of limb functional activities between the THA and HA groups. However, the THA group incurred higher surgical trauma and total hospitalization costs. No significant difference was observed between the THA and HA groups in terms of postoperative time to ambulation (14.430 versus [vs.] 13.163 days; *P* =  0.803), length of hospitalization (11.187 vs. 10.737 days; *P* =  0.777), VAS score 1 month after surgery (2.333 vs. 2.360; *P* =  0.933), and Harris score 1 month after surgery (81.958 vs. 83.278; *P* =  0.379). Patients in the THA group exhibited significantly longer operative duration (157.08 vs. 85.08 min; *P* =  0.000), more frequent occurrences of longer surgical incisions (14.604 vs. 9.573 cm; *P* =  0.000), higher blood loss (465.63 vs. 326.23 ml; *P* =  0.000), greater need for blood transfusions (235.416 vs. 112.295 ml; *P* =  0.017), reductions in hemoglobin (38.583 vs. 25.344 g/L; *P* =  0.000) and albumin (14.687 vs. 10.819 g/L; *P* =  0.000) levels, as well as higher total surgical costs (vs.; *P* =  0.000).

**Table 2:** Surgery-related data of patients in two groups.

	THA (n = 48)	HA (n = 61)	*t* or Z	*p* value	Cohen’s d	95%CI
Operation time (*x ± s*, min)	157.08 ± 37.315	85.08 ± 18.585	12.282	0.000	0.773	72.001(61.217,82.786)
Incision length (*x ± s*, cm)	14.604 ± 2.200	9.573 ± 2.061	12.279	0.000	0.763	5.03040(4.21827, 5.84253)
Blood loss (*x ± s*, ml)	465.63 ± 225.774	326.23 ± 176.920	3.615	0.000	0.325	139.395(62.954, 215.8370
Blood transfusion (*x ± s*, ml)	235.416 ± 304.393	112.295 ± 196.141	2.433	0.017	0.234	123.12158 (27.67494, 218.56823)
Hemoglobin drop (*x ± s*, g/L)	38.583 ± 11.917	25.344 ± 12.124	5.702	0.000	0.482	13.23907(8.63624, 17.84191)
Albumin drop (*x ± s*, g/L)	14.687 ± 3.102	10.819 ± 4.1813	5.542	0.000	0.465	3.86783(2.43511, 5.30055)
Postoperative time to ambulation (*x ± s*, d)	14.430 ± 27.144	13.163 ± 25.745	0.250	0.803	0.024	1.27357(−8.81224, 11.35937)
Hospitalization days (*x ± s*, d)	11.187 ± 5.421	10.737 ± 9.862	0.284	0.777	0.028	.44980(−2.69148, 3.59107)
Total cost (*x ± s*, ¥)	61875 ± 17938	49295 ± 11688	4.417	0.000	0.384	1.25799(0.69333, 1.82265)
Postoperative VAS score (*x ± s*)	2.333 ± 1.717	2.360 ± 1.663	−0.084	0.933	0.008	-.02732(−0.67280, 0.61816)
Harris score (*x ± s*)	81.958 ± 6.915	83.278 ± 8.352	−0.882	0.379	0.086	−1.32036(−4.28635, 1.64564)

THA: Total hip arthroplasty

HA: Hemiarthroplasty

VAS: visual analogue scale

### 3.3 Data from the THA and HA groups during the 5-year follow-up

**Fig 4:**
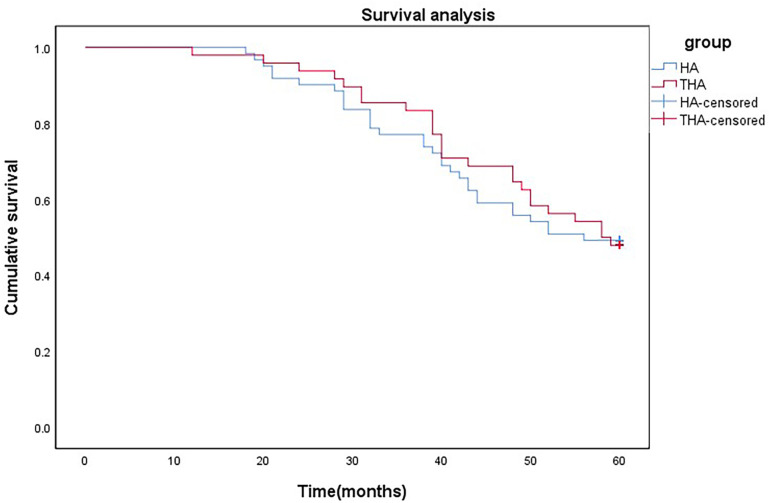
Survival analysis of two groups of patients.

Characteristics of the 2 groups of patients 5 years after surgery are summarized in Table 3. Statistical analysis indicated no significant differences in 5-year postoperative complication rates, treatment satisfaction, or patient survival rates between the THA and HA groups. In the fifth year after surgery, there were 23 and 30 survivors in the THA and HA groups, respectively, corresponding to survival rates of 47.91% and 49.18%, respectively. Patient satisfaction with surgery was 19 of 23 (82.60%) and 25 of 30 (83.33%) in the THA and HA groups, respectively. Seven patients in the THA group and 10 in the HA group experienced complications. Two patients in each group underwent revision surgery. There were no significant differences in the 5-year postoperative survival rate (*P* =  0.896), satisfaction (*P* =  0.945), complication rate (*P* =  0.796), or revision surgery rate (*P* =  0.807) between the groups. Survival analysis revealed no statistical difference in 5-year survival rates between the 2 groups (χ^2^ =  0.029; P =  0.865) (Fig 4).

**Table 3:** Five-year follow-up data on the surgical efficacy of patients in two groups.

	THA (n = 48)	HA (n = 61)	*t or χ2*	*p* value	Phi	OR(95%CI)
Survival	23	30	0.017	0.896	−0.013	0.951(0.446,2.026)
Satisfaction	19/23	25/30	0.005	0.945	−0.010	0.950(0.224,4.025)
Complications	7	10	0.067	0.796	−0.025	0.871(0.305,2.488)
Revisions	2	2	0.060	0.807	0.023	1.283(0.174,9.454)

THA: Total hip arthroplasty

HA: Hemiarthroplasty

OR: Odds Ratio

## 4. Discussion

The optimal treatment option for older patients who experience FNF remains controversial [27], particularly for those 70–85 years of age. Limited comparative research has investigated the efficacy of THA versus HA in patients with FNF and sarcopenia. This study found no statistically significant differences in the postoperative time to ambulation, length of hospitalization, postoperative VAS scores, and postoperative Harris scores between the THA and HA groups. Moreover, there was no significant difference in patient survival rate, functional satisfaction with the affected limb, complication rate, or re-revision surgery rate 5 years after surgery. Notably, the HA group benefitted from advantages such as shorter operative duration, smaller incision length, reduced blood loss, decreased need for blood transfusion, lower decline in hemoglobin and albumin levels, and lower total hospitalization costs. For patients with FNF and sarcopenia, both THA and HA yield similar short- and long-term surgical effects; however, HA is associated with less trauma and lower costs.

The hip joint consists of the femoral head, femoral neck, and acetabulum, and serves as an important connection between the lower limbs and trunk. In cases of FNF, blood supply to the femoral neck is disrupted, making fracture healing difficult. Consequently, elderly patients often require artificial joint replacement, which reduces the risk for mortality and complications associated with non-surgical treatments, including pressure ulcers, deep vein thrombosis in the lower extremities, lung infections, and reduced limb function [30]. Theoretically, THA provides a perfect fit between the acetabular and femoral prostheses, enhancing joint stability, and reducing friction. This results in faster and better recovery of joint function as well as a longer lifespan for the prosthesis. However, THA is associated with greater trauma, longer operative duration, and increased blood loss and surgical costs, due to the need for additional acetabular reconstruction. Similar to our study, Luo et al. [27] found that patients undergoing THA experienced significantly longer operative durations and greater blood loss. However, the 5-year follow-up indicated that THA resulted in improved hip function, higher Harris score, and reduced pain. The discrepancies between these findings and our results may be attributed to variations in follow-up duration, particularly concerning the influence of comorbid sarcopenia. A study from the United States found that patients undergoing THA experienced greater bleeding than those who underwent HA. However, the difference in bleeding volume was minimal and not clinically significant [31]. This finding contrasts with our results, which may have been influenced by factors such as choice of surgical method, surgical technique, use of hemostatic agents, such as tranexamic acid, and the presence of sarcopenia. Therefore, THA is more suitable for patients with greater activity requirements and stronger bodies [32]. In contrast, HA causes less trauma, has a shorter operative duration, and is more suitable for elderly patients with lower tolerance levels [33]. An Australian study found that, compared with HA, patients undergoing THA had a shorter overall length of hospital stay, were more likely to be discharged directly home, and were less likely to require admission to an aged care facility. In contrast, our study revealed no significant difference in the length of stay between the THA and HA groups [34]. On analyzing the reasons for this discrepancy, factors such as differences in sample size, variations in patient treatment processes, rehabilitation methods, and the presence of sarcopenia may be relevant.

Previous studies have indicated that THA is associated with longer operative duration and greater blood loss than HA [35,36], findings consistent with those of the present study. Interestingly, a recent study revealed that the risk for 12-month dislocation was significantly higher in elderly patients with FNF who underwent THA [37]; however, this increase did not affect the risk for revision/conversion. Luo et al. [27] found no statistically significant differences in the incidence of complications or dislocation rates between 2 groups of patients. Although our study did not specifically evaluate the risk for dislocation, we found no significant differences in overall complication rates between the 2 groups.

As the global population ages, the prevalence of frailty and sarcopenia will increase. Among various types of fractures, hip fractures are, unfortunately, highly debilitating [38,39]. Sarcopenia, which is characterized by a decline in muscle mass and strength, commonly occurs with advanced age [40]. The pathophysiology of sarcopenia is complex and may involve cellular aging, malnutrition, lack of exercise, tissue inflammation, hormonal imbalance, and reduced blood supply [41]. At the cellular level, sarcopenia is evident through reduced mitochondrial function, decreased muscle protein synthesis, and increased catabolism [42,43].

Sarcopenia has been identified as an independent risk factor for non-osteoporotic vertebral fractures [44]. Frailty increases the risks for falls and fractures, and vice-versa, which further increase frailty itself [45]. In a study involving patients <  70 years of age who underwent internal fixation for FNF, 10% had osteoporosis and 33% had sarcopenia after 10 years of follow-up [46]. Therefore, it is crucial to promote regular muscle-sparing resistance training for individuals who experience FNF. Notably, this study found no statistically significant differences in age, sex, SMI, or BMI between the THA and HA groups. This helps minimize the risk for bias caused by patient nutritional status. Hemoglobin and albumin levels in both groups decreased to varying extents, with a more pronounced decrease observed in the THA group. This reflects the physical strain on patients’ bodies due to fractures and surgical trauma. Combined with the advantages of other surgery-related indicators in the HA group, HA offers the benefits of reduced trauma and lower surgical costs. It also demonstrates surgical efficacy comparable with THA and is considered to be a more suitable surgical option for those with FNF and sarcopenia. Previous studies have reported reoperation rates of 35%–47% in elderly patients with FNF [47,48]. In contrast, patients in the present study had a lower rate of revision surgery. This difference can be attributed to variations in the scope of surgery. While this study specifically addressed prosthetic revision surgery, the aforementioned studies encompassed all surgeries related to the hip joint, such as refractures and joint prosthesis dislocations.

Currently, effective treatments for sarcopenia are lacking, thus prompting an urgent need for further research. The primary approach to treating sarcopenia involves non-pharmacological means. Adequate nutrition, including amino acids, proteins, calcium, and vitamin D, is crucial. Promising strategies involve combining nutritional supplementation with various exercises, such as resistance, aerobic, and balance training [18]. Although there are no medications that specifically target sarcopenia, testosterone, growth hormones, and beta-adrenergic receptor agonists are commonly used to improve this condition [49]. Hormone replacement therapy enhances muscle mass in older adults [50]. Additionally, anti-osteoporosis treatments may have a synergistic effect in treating sarcopenia due to the close association between the 2 conditions. Some researchers have discovered that erythropoietin can be a potential treatment option for female patients with FNF and sarcopenia because it improves perioperative muscle strength [51]. In a short-term follow-up study, beta-hydroxy-beta-methylbutyric acid (HMB) supplementation alone helped prevent the loss of muscle mass and grip strength in older hip-replacement patients with sarcopenia. Resistance training was also found to effectively improve muscle mass and grip strength in patients, regardless of HMB supplementation [52]. Importantly, the treatment of sarcopenia requires interdisciplinary collaboration among orthopedics, geriatrics, nutrition, and rehabilitation medicine.

## 5. Limitations

The present study had some limitations, the first of which was its retrospective design, which was vulnerable to both selection and information biases, which may have potentially affected the reliability of the results. Some patients were followed-up during the novel coronavirus (i.e., “COVID-19”) pandemic, which may have influenced their physical function and mortality. It is important to note that our study was conducted before there was a consistent decline in surgical costs under government leadership. Furthermore, as joint replacement surgery technology continues to improve, it will also affect the recovery of patient limb function. It is noteworthy that our study did not continuously track patient BMI during fracture recovery, and there was no pharmacological intervention for sarcopenia or inclusion of bone density in the relevant analysis. The clinical data of the patients included in this study were collected prior to 2018. To achieve a more accurate diagnosis, the 2018 EWGSOP sarcopenia diagnostic criteria were employed. However, this approach may introduce sampling bias. Larger sample sizes and multicenter follow-up studies are necessary to further validate our findings.

## 6. Conclusions

Taken together, for patients 70–85 years of age with FNF and sarcopenia, the surgical outcomes of HA were comparable with those of THA. However, HA is less invasive and was associated with lower hospitalization costs, making it the preferred surgical option. Further analysis of clinical samples from additional centers is necessary to establish reliable clinical guidelines.

## Abbreviations

BMI: Body mass index
CT: Computed tomography
EWGSOP: European Working Group on Sarcopenia in Older People
FNF: Femoral neck fracture
HA: Hemiarthroplasty
HMB: beta-hydroxy-beta-methylbutyric acid
THA: Total hip arthroplasty
SMI: Skeletal muscle index
VAS: Visual analog scale.
